# Glutathione *S-*Transferase P1, Maternal Smoking, and Asthma in Children: A Haplotype-Based Analysis

**DOI:** 10.1289/ehp.10655

**Published:** 2007-12-12

**Authors:** Yu-Fen Li, W. James Gauderman, David V. Conti, Pi-Chu Lin, Edward Avol, Frank D. Gilliland

**Affiliations:** 1 Department of Preventive Medicine, Keck School of Medicine, University of Southern California, Los Angeles, California, USA; 2 Institute of Environmental Health, China Medical University, Taichung, Taiwan

**Keywords:** asthma, children, *GSTP1*, haplotypes

## Abstract

**Background:**

Glutathione *S*-transferase P1 (*GSTP1*) plays a role in a spectrum of respiratory diseases; however, the effects of sequence variation across the entire locus in asthma pathogenesis have yet to be determined.

**Objectives:**

This study was designed to investigate whether sequence variations in the *GSTP1* coding and promoter regions are associated with asthma and wheezing outcomes and to determine whether variants affect susceptibility to maternal smoking.

**Methods:**

Four haplotype tagging SNPs were selected that accounted for 83% of the common haplotypic variation in *GSTP1*. The associations of *GSTP1* variants with asthma and wheezing were assessed among white children in the Children’s Health Study (CHS).

**Results:**

The Ile105Val allele and a SNP in the upstream promoter region (SNP1: rs6591255, putative transcription factor 1 binding site) were associated with asthma and wheezing outcomes, an association observed in two cohorts of the CHS recruited in different years. Haplotypes that included both the promoter SNP (i.e., rs6591255) and the 105 Val variant were associated with an increased risk for asthma in non-Hispanic whites. Using SNP- and haplotype-based approaches, the effect of maternal smoking on wheezing was largest in children with the Ile105Val allele.

**Conclusions:**

Variants in both the promoter and coding regions of the *GSTP1* locus may contribute to the occurrence of childhood asthma and wheezing and may increase susceptibility to adverse effects of tobacco-smoke exposure.

Over the last 25 years, asthma has emerged as an increasingly important public health problem (Anderson et al. 1994; Rappaport and Boodram 1998). The pathogenesis and etiology of asthma is complex and not fully understood. Although a rapid rise in childhood asthma prevalence suggests a role for environmental factors in the etiology of this evolving epidemic, genetic susceptibility to environmental stressors is also likely to influence the occurrence of asthma (Blumenthal 2005; Cookson 2002; Hemminki et al. 2007).

Evidence suggests that members of the glutathione *S*-transferase (GST) superfamily contribute to asthma pathogenesis. We have previously reported that *GSTM1* is associated with asthma risk in children exposed to tobacco smoke (Gilliland et al. 2002b). The GST superfamily includes a number of additional candidate susceptibility genes for asthma because several members, including *GSTP1,* are expressed in the respiratory tract and function in processes implicated in asthma pathogenesis, including oxidant defenses, xenobiotic metabolism, and detoxification of hydroperoxides (Strange et al. 2001). DNA sequence variants in the *GSTP1* locus may contribute to susceptibility to oxidative stress and airway inflammation, which are key processes in asthma pathogenesis. A functional sequence variant in *GSTP1* at codon 105 (Ile105Val) has been associated with asthma and susceptibility to the adverse effects of tobacco smoke and ambient air pollution in several studies (Aynacioglu et al. 2004; Ercan et al. 2006; Fryer et al. 2000; Gilliland et al. 2002a, 2004; Hemmingsen et al. 2001; Lee et al. 2004, 2005; Mapp et al. 2002; Palmer et al. 2006; Spiteri et al. 2000; Tamer et al. 2004). Although evidence indicates a role of the GSTP1 Ile105Val variant in asthma, the effect of other common variants in *GSTP1* coding and promoter region and the joint effects with tobacco-smoke exposure have yet to be fully determined.

The human genome is characterized by regions of strong linkage disequilibrium (LD) that have been labeled as haplotype blocks by Gabriel et al. (2002). The common genetic variation across the region can be predicted using only a subset of all single nucleotide polymorphisms [haplotype tagging SNPs (htSNPs)] (Stram et al. 2003). These can be used to impute haplotypes and provide an indirect assessment of potential common causal variants that may not have been genotyped. To further investigate the effect of common *GSTP1* variants to asthma occurrence and susceptibility to tobacco smoke, we examined associations between common *GSTP1* haplotypes, tobacco-smoke exposure, childhood asthma, and related symptoms among participants in the Children’s Health Study.

## Methods

### Study population

The Children’s Health Study (CHS) recruited children from public school classrooms from grades 4, 7, and 10 in 12 Southern California communities. At study entry, in the spring of 1993, a parent or guardian of each participating child completed a self-administered questionnaire on demographics, medical and family health history, smoking exposures, and household characteristics. In the fall of 1995, a second group of fourth-grade students was recruited and completed the same baseline questionnaire as the group enrolled in 1993. A parent or guardian of each participating child provided written informed consent. Details on the design, site selection, subject recruitment, and assessment of health effects have previously been reported (Gilliland et al. 2002b; Peters et al. 1999). This analysis included 3,082 non-Hispanic and Hispanic white children who participated in the genetic studies. Children and a subset of their parents (579 child–parent trios) provided buccal cell specimens as a source of germline DNA for genotyping.

### Outcome assessment: asthma/wheezing

We used questionnaire responses provided by parents or guardians to categorize children’s asthma status, age at asthma diagnosis, and wheezing history as previously described (Gilliland et al. 2002b; Li et al. 2000). Children were classified as having a lifetime diagnosis of asthma if the adult completing the questionnaire reported that a doctor had ever diagnosed the child as having asthma. Asthma onset by 3 years of age was classified as early-onset asthma. Late-onset asthma was defined as age of onset for asthma after 3 years of age. Current wheezing was defined as a positive response to both of the following questions: “Has your child ever wheezed? If yes, has this happened in the past 12 months?” Any medication for wheezing was assessed for the 12 months before the interview.

### Exposure assessment

Exposure assessment methods have been previously reported (Gilliland et al. 2002b; Peters et al. 1999). In brief, exposure to maternal smoking during the *in utero* period, secondhand-smoke exposure, and the number of smokers in the household were collected by questionnaire at study entry. Ambient air pollutants were monitored at central site monitors located in each of the study communities, and average annual exposure levels were computed. Monitored pollutants included ozone, nitrogen dioxide, and particulate matter with an aerodynamic diameter of up to 2.5 μm or 10μm (PM_2.5_ and PM_10_).

### Genotyping

Buccal scrapes were collected using standard protocols, and genomic DNA was isolated using a PURGENE DNA isolation Kit (Gentra Systems, Minneapolis, MN, USA). The polymorphisms were identified using allele-specific MGB probes on an ABI PRISM 7700 Sequence Detector (Applied Biosystems, Foster City, CA, USA). The sequences of primers and probes used are listed in the Supplemental Material, Table 1 (online at http://www.ehponline.org/members/2007/10655/suppl.pdf).

### Characterizing LD and selecting htSNPs

To investigate the LD patterns for *GSTP1*, we examined resequencing data for *GSTP1* in a panel of 90 subjects provided by the National Institute of Environmental Health Sciences (NIEHS) Environmental Genome Project (http://egp.gs.washington.edu/) (NIEHS SNPs 2007) to characterize the haplotype block structure by using Haploview version 3.3 (download from http://www.broad.mit.edu/mpg/haploview/download.php) (Barrett et al. 2006). One sample was not included because only 40% of the SNPs had genotyping results. Twenty-six of the 36 SNPs found in the sequencing were excluded because of a lower minor allele frequency (MAF ≤ 0.20) or a high missing rate (≥25%) among samples.

**Figure 1:**
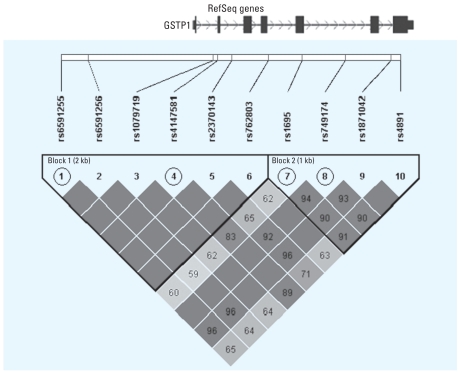
The location and linkage disequilibrium display of 10 resequenced *GSTP1* SNPs. The data are from the NIEHS Environmental Genome Project. The block structure of *GSTP1* is defined using confidence intervals of D′ among SNPs (pairwise 100 × D′ shown in the lozenge-shaped cells, an empty D′ value indicates D′ = 1). Two htSNPs (circled) are selected for each block based on the *R*_h_^2^ > 0.85 criteria.

As shown in Figure 1, two haplotype blocks with substantial interblock LD were defined based on the method using the confidence intervals (CIs) of D′ proposed by Gabriel et al. (2002) (with the upper CI as 0.97 and the lower CI as 0.70). The squared correlation (*R*_h_^2^) between the true haplotypes (h) and their estimates were calculated, and the calculation of *R*_h_^2^ is described in detail by Stram et al. (2003). The htSNPs were then chosen using TagSNPs (download from http://www-rcf.usc.edu/~stram/tagSNPs.html) (Stram 2004). This program implements an expectation maximization (EM) algorithm approach to find the minimum set of SNPs (within a block) that would have *R*_h_^2^ ≥0.85 for all haplotypes with an estimated frequency of ≥ 5%. We selected two htSNPs for each of the two blocks (rs6591255 and rs4147581 for the first block with *R*_h_^2^ = 0.87; rs1695 and rs749174 for the second block with *R*_h_^2^ = 0.91). All four htSNPs accounted for 83% of the haplotypic variation in the *GSTP1* locus without considering the block structure.

### Statistical analyses

#### SNP-based analysis

We first examined the association of each of the four *GSTP1* htSNPs with asthma and wheezing outcomes using multiple logistic regressions. Models were adjusted for *a priori* selected covariates including communities of residence, sex, age, gestational age, and smoke exposure (both *in utero* and secondhand). We used the dominant genetic model to assess the association of each htSNP with asthma and wheezing outcomes. We performed a likelihood ratio test (LRT) to test the global association of four variants in *GSTP1* with asthma and wheezing outcomes. To assess confounding by admixture, we examined genotype associations in child–parent trios using a logistic-regression model that included indicators of parent mating type (Gauderman 2003). In addition, we stratified our study population by non-Hispanic and Hispanic whites (two major ethnic groups) to assess whether associations differed by ethnicity. We formally tested for ethic differences in associations, and if effect estimates and test of significance supported no ethnic differences, the analyses were conducted in the combined group with adjustment for ethnicity.

#### Haplotype-based analysis

Because phase cannot be uniquely defined for subjects that have heterozygous genotypes, haplotype frequencies for each ethnic group (non-Hispanic and Hispanics whites) were separately estimated from the genotype data using the EM algorithm. Following the method of Zaykin et al. (2002) and Wallenstein et al. (1998), we coded the haplotype as the (estimated) number of copies of each haplotype a person carries. For example, if one individual carries one copy of haplotype A and one copy of haplotype B, the coding for haplotype A and B would be one, and the coding for the rest of the haplotypes could be zero. The sum of all number of copies of each haplotype for a person should be two. Except for the most common haplotype, which was the referent haplotype in the analysis, these (estimated) numbers of copies of each haplotype a person carries were used in a logistic model with the same adjustment variables listed above. Thus, the odds ratio (OR) for the outcome represents the increase in risk for a single copy of a particular haplotype relative to the referent haplotype (Wallenstein et al. 1998). The most common haplotype was used as the reference in the haplotype-based analysis. Moreover, likelihood ratio tests with 5 degrees of freedom were performed for the omnibus test of haplotype association. All statistical analyses were carried out by SAS version 9.1 (SAS Institute Inc., Cary, NC, USA), and all tests were two-sided with 0.05 as the significant level.

## Results

The characteristics of participants in this study are described in Table 1. Most children were non-Hispanic white and < 10 years of age. *In utero* exposure to maternal smoking occurred among 19.8% of non-Hispanic whites and 10.7% of Hispanic whites. Personal smoking was rare in both ethnic groups (about 1%). Similar percentages of non-Hispanic whites and Hispanic whites (15.3% and 13.8%, respectively) reported any lifetime diagnosis of asthma. The prevalence of wheezing in the preceding 12 months was 19.9% and 16.8% for non-Hispanic and Hispanic whites, respectively. About 11% of participants took medication for wheezing.

The genotype frequencies of the four *GSTP1* htSNPs are presented in Table 2, and the pairwise measures (D′ and *r*^2^) of linkage disequilibrium (LD) are shown in Table 3. The genotype distributions of the four SNPs were consistent with Hardy–Weinberg equilibrium. The allele frequencies of these SNPs differed in non-Hispanic and Hispanic whites, and the four htSNPs appeared to show stronger LD across the locus in non-Hispanic whites than in Hispanic whites.

In the SNP-based analysis, the four *GSTP1* SNPs jointly explained a statistically significant portion of the occurrence of lifetime asthma and wheezing occurrences (Table 4). The overall pattern of associations was similar in non-Hispanic and Hispanic whites, although the association was statistically significant only in non-Hispanic whites, a finding likely attributable to the larger sample size of non-Hispanic whites. The variant allele of SNP1 was associated with an increased risk for all asthma and wheezing outcomes with no important ethnic differences in the magnitudes of the associations. Carrying one variant allele of SNP2 was protective for asthma and wheezing, although the association reached statistical significance for lifetime asthma and early-onset asthma, again with little evidence for ethnic differences in effects. The variant allele of SNP3 (Ile105Val) was associated with an increased risk of early-onset asthma. SNP4 showed a similar pattern with asthma and wheezing as SNP1.

Based on *a priori* knowledge that SNP1 and SNP3 are not in strong LD (Table 4) and may produce functional differences in the expression and/or enzymatic function, respectively, we fitted reduced models that include only these two SNPs (Table 5). The joint model of the two functional SNPs provided the best fit to these data and showed that carrying a variant allele of SNP1 increased the risk of asthma and wheezing, but having a variant allele of SNP3 did not have a significant association with asthma and wheezing. Because the CHS cohort actually represented two discrete enrollment periods (a fourth-, seventh-, and tenth-grade group recruited in 1993 and an additional fourth-grade contingent in 1996), we examined whether associations were also observable in the two independent groups. The results were consistent in these two groups, and the stronger association of SNP1 compared with SNP3 was observed in the two CHS cohorts [Supplemental Material, Table 2 (online at http://www.ehponline.org/members/2007/10655/suppl.pdf)].

Haplotype frequencies defined using the four *GSTP1* htSNPs are shown in Table 6. The number and frequency of haplotypes varied among non-Hispanic and Hispanic whites. For example, the haplotype carrying the variant allele for SNP3 and common alleles for the other three SNPs (henceforth labeled h0010) was rare in non-Hispanic whites (frequency = 0.008) and relatively common in Hispanic whites (frequency = 0.209). With one exception, we collapsed the rare haplotypes with frequency < 0.05 into a single composite category for data analysis as indicated in Table 6 [leaving only the haplotype that isolates the Ile105Val variant as a separate category (h0010)].

Variation in the upstream region of the locus was associated with asthma and wheezing. Although the omnibus test of haplotype association (by the likelihood ratio test with 5 degrees of freedom) was only statistically significant in the combined group, haplotypes containing the variant allele of SNP1 (i.e., h1000 and h1011) were associated with an increased risk of asthma (Table 7). Compared with having two copies of the most common haplotype (h0100), people with a copy of h1000 were at a 1.3-fold higher risk of lifetime diagnosis of asthma (95% CI, 1.0–1.7). Children with the haplotype h1011 showed an increased risk for the lifetime diagnosis of asthma compared with children carrying the reference haplotype. In contrast, the h0010 haplotype (105Val with no other variants) was associated with a reduced risk for current wheezing. There were no significant ethnic differences in these associations.

We have previously reported that variants in GSTs affect the risk of asthma associated with maternal smoking (Gilliland et al. 2002b). To further investigate the effect of *GSTP1* variants on the relationship between *in utero* exposure to maternal smoking and asthma/wheezing outcomes, we examined the joint effects of the common haplotype and *in utero* exposure to maternal smoking in both ethnic groups combined because there was little evidence for ethnic heterogeneity in associations. Among the common haplotypes shown in Table 6, the effects of maternal smoking on wheezing were significantly larger in those with h0010 compared with the other haplotypes (Table 8). The same pattern of effects was observed in the h1011 haplotype (Table 9). Because the haplotype associations involved the *GSTP1* Ile105Val SNP, we then investigated the relationship between the Ile105Val variant, *in utero* exposure to maternal smoking and asthma/wheezing outcomes (Table 10). Compared with those with no exposure and homozygous Ile 105 genotype, children exposed to *in utero* maternal smoking and carrying at least one variant *GSTP1* Ile105Val allele had about twice the risk of current wheezing and requiring medication for wheeze (OR = 1.9 for both; and 95% CI, 1.3–2.6 and 1.2–2.8, respectively; interaction *p*-value = 0.04 for both). Consistent with an important role of the Val105 variant in the association between *in utero* exposure to maternal smoking and asthma/wheezing, h1011 modified the effect of *in utero* exposure to maternal smoking (Table 9); however, SNP1 and SNP4 did not modify the association in the SNP-based analyses.

To assess the potential for confounding by admixture, we investigated the genetic associations in a cohort subsample of 579 child–parent trios (for both diseased and nondiseased children). The trio analysis showed that the risk estimates were generally consistent with the results for the entire cohort. The point estimates for SNP1 and SNP4 were higher [Supplemental Material, Table 3 (online at http://www.ehponline.org/members/2007/10655/suppl.pdf)], but the confidence-intervals width was about twice as wide in the trio analysis as in the main study, reflecting the smaller sample size of complete trios.

The effect of *GSTP1* on asthma and wheezing did not differ with respect to sex, secondhand-smoke exposure, number of smokers in the household, personal smoking, and ambient air pollutants including ozone, nitrogen dioxide, and PM_2.5_ and PM_10_ (data not shown).

## Discussion

We found that DNA sequence variants in both the promoter and coding regions of the *GSTP1* locus may contribute to the occurrence of childhood asthma and wheezing. The overall evidence is consistent with a complex age-dependent role in the pathogenesis of asthma and wheezing for the *GSTP1* Ile105Val SNP, which affects enzyme function. Furthermore, the effect of *in utero* exposure to maternal smoking on current wheezing and medication for wheeze depends primarily on the *GSTP1* 105Val variant allele both in the haplotype (h0010) and SNP analyses. Our finding that a potentially functional SNP located in the promoter region of *GSTP1* is associated with asthma is novel and suggests that transcriptional regulation may be important in asthma development.

*GSTP1* is located on chromosome 11q13, a previously suggested candidate region for asthma, bronchial hyperresponsiveness, and asthma-associated quantitative traits in some linkage studies (Doull et al. 1996). The mechanism for the effects of *GSTP1* on respiratory illnesses may involve pathways that affect responses to oxidative stress (Ercan et al. 2006; Hayes and Strange 2000). GSTs contribute to protection against oxidative stress by using glutathione to detoxify a variety of electrophilic compounds including oxidized lipid, DNA, and catechol products generated by reactive oxygen species–induced damage to intracellular molecules (Hayes and Strange 1995, 2000). Because *GSTP1* is strongly expressed in the respiratory epithelium and is the dominant GST in the lung, variation in *GSTP1* function may have larger effects on respiratory health outcomes than do other members of the GST superfamily. The role of *GSTP1* protecting against reactive oxygen species and their secondary products may be mediated by the 105 variant (Peterhans 1997).

To our knowledge, the association between SNP1 (rs6591255) with asthma and wheezing has not been previously reported. Based on the SNP- and haplotype-based analyses in the present study, such association of SNP1 is not attributable to the LD with the *GSTP1* Ile105Val allele. This SNP is located in a putative transcription factor 1 (TCF-1) binding site upstream of the transcription start site and may potentially be a functional SNP. However, the role of TCF-1 in asthma pathogenesis has yet to be determined. The promoter region is complex and could harbor other functional variants in LD with SNP1. Cauchi et al. (2006) completely sequenced the promoter region of the *GSTP1* gene in 40 Euro-Caucasian individuals and reported that one common haplotype comprising the common allele of the SNP1 in the present study interacts with chemopreventive exposure in luciferase reporter constructs. This novel finding is consistent with our finding that SNP1 is an upstream SNP important in reducing the risk of asthma and wheezing symptoms. The role of the promoter region of *GSTP1* needs further study to verify whether the association reported here is attributable to LD with another functional SNP or its own functionality as a TCF-1 binding site.

Our study has some potential limitations that may influence the interpretation of our results. We recognize that asthma is a complex disease, and studying only the *GSTP1* gene does not provide a complete picture of genetic susceptibility. Interaction between *GSTP1* and *GSTM1* has been reported for xenobiotic enhancement of allergic responses (Gilliland et al. 2004) and on childhood asthma risk (Lee et al. 2005). In the present study, there is insufficient power to assess higher-order interactions between *GSTP1* SNPs and haplotypes with other genes and smoking.

Selection bias is a common concern in cross-sectional studies. The group of children with genotyping data included in this analysis did not differ substantially from those without genotyping data on a broad range of demographic, medical history, and household exposure factors [Supplemental Material, Table 4 (online at http://www.ehponline.org/members/2007/10655/suppl.pdf)]. Therefore, selection bias is an unlikely explanation for our findings. Another possible limitation might be recall bias with regard to early life events. To evaluate this possibility, we resurveyed 691 study participants about the mother’s smoking history during pregnancy and about asthma status in a counter-matched case–control study nested in the CHS (Li et al. 2005) as we tried to investigate the dose effect of smoking. The kappa coefficients for the agreement between the two repeated measurements were 0.80 and 0.75 for asthma and smoking, respectively. The agreement is acceptable, especially as the agreement of smoking was not statistically significantly different by asthma status. We assessed recall of asthma status in the same nested case–control study and found that the concordance of parental reports of asthma and medical records’ documentation of asthma was good. Based on the 2001 National Health Interview Survey, it was estimated that 113.4 per 1,000 Americans had been diagnosed with asthma by a physician in their lifetime, and, among children 5–17 years of age, the lifetime prevalence was 144.2 per 1,000 which was close to the prevalence of asthma in the present study (American Lung Association 2006). Moreover, we observed the same results when we excluded early transient wheezing cases [Supplemental Material, Table 5 (online at http://www.ehponline.org/members/2007/10655/suppl.pdf)]. Population stratification can be a concern in genetic association studies. In our investigation, although the estimates were more uncertain because of a smaller sample size in the trio analyses, the case–control and complete trio analyses indicate that confounding by population substructure is unlikely to explain our findings.

Some of our results were based on a haplotype approach. The selection of htSNPs was based on 90 individual genotypes provided by the NIEHS Environmental Genome Project. Although haplotype analysis is very dependent on the definition of blocks, it was not an issue for the present study because the four SNPs selected for this study represented > 80% of the general population’s variation. The EPG data did not carry ethnicity identifiers, so we could not stratify the data when selecting htSNPs. However, we assessed how well the four selected htSNPs represented the haplotype diversities in two other ethnically identified subgroups, each containing 70 individuals. The *R*_h_^2^ was 0.76 and 0.71 respectively for non-Hispanic and Hispanic whites, implying that the four selected htSNPs had good coverage for the two ethnic groups in the present study. Nevertheless, the ability to detect a true disease variant using a haplotype approach can be attenuated if the variant is distributed across several haplotypes in the population and the Ile105Val allele is split into two major haplotypes in Hispanic whites. In our analyses, we adopted the commonly used approach of clustering rare haplotypes into a composite category. However, this composite category likely included one or more haplotypes carrying the disease variant, thereby reducing the analytical power to contrast this variant against other haplotypes.

In summary, the *GSTP1* locus is associated with asthma and wheezing outcomes in children. Both the well-studied Ile105Val variant and an upstream SNP are associated with the risk of asthma; however, the effects appear to occur in opposite directions. This pattern may explain some of the inconsistencies in previous studies of the 105 variant and asthma. The 105Val allele variant appears to modulate the effects of *in utero* exposure to maternal smoking on asthma and wheezing. Additional studies of sufficient size are needed to independently replicate and expand our findings to investigate sequence variation in the promoter region of the *GSTP1* gene and the interaction of *GSTP1* haplotypes with other genes and environmental/endogenous sources of reactive oxygen species.

## Figures and Tables

**Table 1:** Selected characteristics of non-Hispanic and Hispanic white CHS participants.^a^

Demographic characteristics/respiratory outcomes	Non-Hispanic white (*n* = 2,122)	Hispanic white (*n* = 960)
Demographic characteristics		
Percent female	51.7	56.8
Age at study entry [years (%)]		
8– < 10	50.5	55.8
10– < 12	21.4	22.2
12– < 14	15.5	11.7
14–18	12.5	10.3
Gestational age (%)		
Full term	87.9	91.2
< 4 weeks early	7.3	6.0
≥4 weeks early	4.7	2.8
With insurance (%)	88.3	73.9
Tobacco-smoke exposure (%)		
*In utero*	19.8	10.7
Ever SHS	36.2	26.9
Current SHS	19.2	12.9
Personal smoking	1.1	0.8
Respiratory outcomes (%)		
Lifetime diagnosis of asthma	15.3	13.8
Early-onset asthma	7.1	6.9
Late-onset asthma	8.2	6.9
Current wheezing	19.9	16.8
Medication for wheeze	11.4	10.2

SHS, secondhand smoke.

a Participants with genotyping data.

**Table 2:** Genotype frequencies of four *GSTP1* htSNPs in non-Hispanic and Hispanic white participants in the CHS.^a^

		Non-Hispanic white		Hispanic white	
*GSTP1* genotype		No.	%	No.	%
SNP1	TT	741	34.9	468	48.8
(rs6591255)	TA	1,024	48.3	399	41.6
	AA	357	16.8	93	9.7
Minor allele (A) frequency	0.41		0.31	
SNP2	CC	527	24.8	367	38.2
(rs4147581)	CG	1,083	51.0	456	47.5
	GG	512	24.1	137	14.3
Minor allele (G) frequency	0.50		0.38	
SNP3	AA	945	44.5	279	29.1
(rs1695)	AG	944	44.5	504	52.5
	GG	233	11.0	177	18.4
Minor allele (G) frequency	0.33		0.45	
SNP4	CC	948	44.7	539	56.2
(rs749174)	CT	948	44.7	362	37.7
	TT	226	10.7	59	6.2
Minor allele (T) frequency		0.33		0.25	

a The genotype distributions of the four SNPs are all in the Hardy–Weinberg equilibrium in both ethnic groups.

**Table 3:** Pairwise measures of linkage disequilibrium (*r*^2^^a^ and D′^b^) for the *GSTP1* htSNPs in non-Hispanic and Hispanic white CHS participants.

D′ \ *r*^2^	SNP1 (rs6591255)	SNP2 (rs4147581)	SNP3 (rs1695)	SNP4 (rs749174)
Non-Hispanic white				
SNP1		0.668^a^	0.550^a^	0.633^a^
SNP2	0.988^b^		0.406^a^	0.448^a^
SNP3	0.875^b^	0.910^b^		0.820^a^
SNP4	0.944^b^	0.961^b^	0.910^b^	
Hispanic white				
SNP1		0.269^a^	0.155^a^	0.625^a^
SNP2	1.000^b^		0.433^a^	0.162^a^
SNP3	0.534^b^	0.935^b^		0.307^a^
SNP4	0.906^b^	0.889^b^	0.862^b^	

a *r*^2^: upper triangle area.

b D′: lower triangle area.

**Table 4:** The association [OR (95% (CI)] of four *GSTP1* htSNPs^a^ with asthma and wheezing in CHS participants by single SNP models.

Ethnicity and outcome	SNP1	SNP2	SNP3	SNP4	LRT^b^*p*-Value
Non-Hispanic + Hispanic white^c^					
Lifetime diagnosis of asthma	1.4 (1.1–1.7)	0.8 (0.6–0.9)	1.2 (1.0–1.5)*	1.3 (1.0–1.5)*	0.022
Early-onset asthma	1.4 (1.0–1.8)*	0.7 (0.5–0.9)	1.3 (0.9–1.8)	1.4 (1.0–1.8)*	0.142
Late-onset asthma	1.4 (1.0–1.9)*	0.8 (0.6–1.0)	1.2 (0.8–1.5)	1.2 (0.9–1.6)	0.109
Current wheezing	1.3 (1.0–1.6)*	1.0 (0.7–1.1)	1.1 (0.8–1.2)	1.2 (1.0–1.4)*	0.027
Medication for wheeze	1.6 (1.2–2.0)	0.9 (0.7–1.2)	1.2 (0.9–1.5)	1.4 (1.1–1.7)	0.004
Non-Hispanic white^d^					
Lifetime diagnosis of asthma	1.5 (1.1–1.9)	0.8 (0.6–1.0)	1.2 (0.9–1.5)	1.2 (0.9–1.5)	0.032
Early-onset asthma	1.5 (1.0–2.2)*	0.8 (0.5–1.1)	1.4 (1.0–2.0)*	1.4 (0.9–1.9)	0.209
Late-onset asthma	1.5 (1.0–2.0)*	0.8 (0.5–1.0)	1.1 (0.7–1.4)	1.1 (0.8–1.5)	0.090
Current wheezing	1.4 (1.1–1.7)	0.9 (0.7–1.2)	1.1 (0.8–1.3)	1.2 (0.9–1.4)	0.031
Medication for wheeze	1.6 (1.2–2.2)	0.9 (0.6–1.2)	1.2 (0.9–1.6)	1.3 (0.9–1.7)	0.024
Hispanic white^d^					
Lifetime diagnosis of asthma	1.3 (0.8–1.9)	0.7 (0.4–1.0)	1.3 (0.8–2.0)	1.5 (1.0–2.1)*	0.143
Early-onset asthma	1.1 (0.6–1.8)	0.6 (0.3–1.0)	1.1 (0.6–1.9)	1.3 (0.7–2.2)	0.236
Late-onset asthma	1.4 (0.8–2.4)	0.8 (0.4–1.3)	1.5 (0.8–2.8)	1.6 (0.9–2.6)	0.469
Current wheezing	1.2 (0.8–1.7)	1.0 (0.6–1.3)	1.0 (0.7–1.5)	1.5 (1.0–2.1)*	0.108
Medication for wheeze	1.5 (0.9–2.4)	1.0 (0.6–1.5)	1.1 (0.7–1.8)	1.7 (1.0–2.6)*	0.186

a SNP1 (rs6591255): T→A; SNP2 (rs4147581): C→G; SNP3 (rs1695): A→G; and SNP4 (rs749174): C→T.

b LRT: comparing the model with the four *GSTP1* htSNPs with the model without the four *GSTP1* htSNPs.

c Models are adjusted for communities of residence, sex, age, gestational age, smoke exposure, and Hispanic ethnicity.

d Models are adjusted for communities of residence, sex, age, gestational age, and smoke exposure.

* *p* < 0.05 although the confidence upper/lower limit is 1.0.

**Table 5:** Multiple logistic-regression models [OR (95% CI)] considering two potential functional *GSTP1* SNPs.^a^

Ethnicity and outcome	SNP1	SNP3	LRT^b^*p*-Value
Non-Hispanic + Hispanic white^c^			
Lifetime diagnosis of asthma	1.4 (1.0–1.8)*	1.0 (0.7–1.3)	0.008
Early-onset asthma	1.2 (0.8–1.7)	1.2 (0.8–1.7)	0.090
Late-onset asthma	1.5 (1.0–2.1)*	0.9 (0.6–1.2)	0.033
Current wheezing	1.5 (1.1–1.8)	0.9 (0.6–1.0)	0.009
Medication for wheeze	1.7 (1.2–2.3)	0.9 (0.6–1.1)	0.001
Non-Hispanic white^d^			
Lifetime diagnosis of asthma	1.6 (1.1–2.3)	0.9 (0.6–1.2)	0.009
Early-onset asthma	1.3 (0.7–2.2)	1.2 (0.7–1.9)	0.067
Late-onset asthma	1.8 (1.1–2.9)	0.7 (0.4–1.1)	0.030
Current wheezing	1.7 (1.2–2.3)	0.8 (0.5–1.0)	0.005
Medication for wheeze	1.9 (1.2–2.8)	0.8 (0.5–1.2)	0.004
Hispanic white^d^			
Lifetime diagnosis of asthma	1.2 (0.8–1.8)	1.2 (0.7–1.9)	0.277
Early-onset asthma	1.1 (0.6–1.9)	1.1 (0.5–1.9)	0.886
Late-onset asthma	1.3 (0.7–2.2)	1.4 (0.7–2.6)	0.233
Current wheezing	1.2 (0.8–1.7)	1.0 (0.6–1.4)	0.622
Medication for wheeze	1.6 (0.9–2.5)	1.0 (0.5–1.6)	0.162

a SNP1 (rs6591255): T→A; SNP3 (rs1695): A→G.

b LRT: comparing the model with the two *GSTP1* SNPs with the model without the *GSTP1* SNPs.

c Models are adjusted for communities of residence, sex, age, gestational age, smoke exposure, and Hispanic ethnicity.

d Models are adjusted for communities of residence, sex, age, gestational age, and smoke exposure.

* *p* < 0.05 although the confidence upper/lower limit is 1.0.

**Table 6:** Haplotype frequencies of four *GSTP1* htSNPs in non-Hispanic and Hispanic white participants in the CHS.

Non-Hispanic white		Hispanic white	
Haplotype^a^	Frequency	Haplotype^a^	Frequency
0100	0.4804	0100	0.3687
1011	0.3012	1011	0.2188
1000	0.0832	0010	0.2087
0000	0.0832	0000	0.0990
0010	0.0078	1000	0.0670
1001^b^	0.0173^b^	1001^b^	0.0148^b^
0110^b^	0.0083^b^	0111^b^	0.0081^b^
1010^b^	0.0055^b^	0011^b^	0.0046^b^
0111^b^	0.0055^b^	1010^b^	0.0041^b^
0011^b^	0.0033^b^	0001^b^	0.0029^b^
0001^b^	0.0021^b^	0110^b^	0.0026^b^
1100^b^	0.0010^b^	0101^b^	0.0008^b^
1110^b^	0.0008^b^		
1101^b^	0.0005^b^		

a 1: minor allele and 0: common allele, by the order of SNP1 (rs6591255): T → A; SNP2 (rs4147581): C → G; SNP3 (rs1695): A → G; and SNP4 (rs749174): C → T.

b Haplotypes were collapsed into a single composite category in the haplotype analyses.

**Table 7:** The association [OR (95% CI)] of common *GSTP1* haplotypes with asthma and wheezing in CHS participants.

Ethnicity and haplotype	Lifetime diagnosis of asthma	Early-onset asthma	Current wheezing	Medication for wheeze
All white^a^				
h0100^b^	1.0	1.0	1.0	1.0
h0000^b^	1.1 (0.8–1.4)	1.1 (0.7–1.6)	1.1 (0.8–1.4)	1.0 (0.7–1.4)
h0010^b^	0.9 (0.7–1.3)	1.2 (0.8–1.8)	0.7 (0.5–0.9)	0.7 (0.5–1.0)
h1000^b^	1.3 (1.0–1.7)	1.2 (0.8–1.8)	1.1 (0.9–1.4)	1.3 (0.9–1.8)
h1011^b^	1.2 (1.0–1.5)	1.3 (1.0–1.7)	1.1 (0.9–1.3)	1.2 (1.0–1.4)
Others	1.0 (0.7–1.6)	1.3 (0.8–2.2)	1.0 (0.7–1.5)	1.0 (0.6–1.6)
*p*-Value of LRT^c^	0.059	0.253	0.055	0.045
Non-Hispanic white^d^				
h0100^b^	1.0	1.0	1.0	1.0
h0000^b^	0.9 (0.7–1.3)	0.8 (0.4–1.4)	1.1 (0.8–1.5)	1.0 (0.7–1.5)
h0010^b^	0.7 (0.2–2.3)	1.8 (0.5–5.9)	0.7 (0.3–2.0)	0.5 (0.1–2.4)
h1000^b^	1.4 (1.1–2.0)	1.2 (0.8–1.9)	1.2 (0.9–1.6)	1.4 (0.9–2.0)
h1011^b^	1.2 (1.0–1.5)	1.3 (0.9–1.7)	1.1 (0.9–1.4)	1.2 (0.9–1.5)
Others	0.9 (0.6–1.5)	1.2 (0.6–2.1)	0.9 (0.6–1.4)	0.9 (0.5–1.5)
*p*-Value of LRT^c^	0.058	0.296	0.408	0.215
Hispanic white^d^				
h0100^b^	1.0	1.0	1.0	1.0
h0000^b^	1.4 (0.9–2.2)	1.6 (0.9–3.1)	1.0 (0.6–1.5)	0.9 (0.5–1.6)
h0010^b^	1.1 (0.7–1.6)	1.3 (0.7–2.2)	0.8 (0.5–1.1)	0.8 (0.5–1.3)
h1000^b^	1.0 (0.5–1.8)	1.2 (0.5–2.6)	0.9 (0.6–1.6)	1.2 (0.7–2.2)
h1011^b^	1.4 (1.0–2.1)	1.4 (0.8–2.4)	1.1 (0.8–1.6)	1.2 (0.8–1.9)
Others	1.6 (0.8–3.3)	2.0 (0.8–5.1)	1.4 (0.7–2.8)	1.4 (0.6–3.2)
*p*-Value of LRT^c^	0.257	0.407	0.460	0.501

a Models are adjusted for communities of residence, sex, age, gestational age, smoke exposure, and Hispanic ethnicity.

b 1: minor allele and 0: common allele, by the order of SNP1 (rs6591255): T → A; SNP2 (rs4147581): C → G; SNP3 (rs1695): A → G; and SNP4 (rs749174): C → T.

c LRT (likelihood ratio test): the omnibus test of haplotype association.

d Models are adjusted for communities of residence, sex, age, gestational age, and smoke exposure.

**Table 8:** Joint effect of *in utero* exposure [OR (95% CI)] to maternal smoking and *GSTP1* haplotype h0010 with asthma and wheezing outcomes, non-Hispanic and Hispanic whites combined.^a^

		*In utero* exposure to maternal smoking	
Outcome	h0010	No	Yes
Lifetime diagnosis of asthma	No	1.0	1.1 (0.7–1.6)
	Yes	1.2 (0.9–1.5)	1.4 (0.9–2.0)
Early-onset asthma	No	1.0	1.7 (0.9–2.9)
	Yes	1.4 (0.9–1.9)	2.0 (1.1–3.3)
Late-onset asthma	No	1.0	0.7 (0.3–1.3)
	Yes	1.1 (0.8–1.5)	1.0 (0.6–1.7)
Current wheezing^b^	No	1.0	1.2 (0.7–1.7)
	Yes	0.9 (0.7–1.1)	1.9 (1.3–2.6)
Medication for wheeze^b^	No	1.0	0.9 (0.5–1.5)
	Yes	1.1 (0.8–1.3)	1.9 (1.2–2.8)

a Models are adjusted for ethnicity, communities of residence, sex, age, gestational age, and secondhand-smoke exposure.

b Significant interaction between *in utero* exposure to maternal smoking and *GSTP1* haplotype h0010.

**Table 9:** Joint effect of *in utero* exposure [OR (95% CI)] to maternal smoking and *GSTP1* haplotype h1011 with asthma and wheezing outcomes, non-Hispanic and Hispanic whites combined.^a^

		*In utero* exposure to maternal smoking	
Outcome	h1011	No	Yes
Lifetime diagnosis of asthma	No	1.0	1.2 (0.8–1.7)
	Yes	1.3 (1.0–1.6)	1.5 (0.9–2.1)
Early-onset asthma	No	1.0	1.7 (1.0–2.8)
	Yes	1.4 (1.0–1.9)	1.9 (1.0–3.1)
Late-onset asthma	No	1.0	0.8 (0.4–1.4)
	Yes	1.3 (0.9–1.7)	1.2 (0.6–1.9)
Current wheezing	No	1.0	1.4 (0.9–2.0)
	Yes	1.1 (0.9–1.3)	2.1 (1.4–3.0)
Medication for wheeze	No	1.0	1.3 (0.8–2.1)
	Yes	1.4 (1.0–1.7)	2.0 (1.2–3.1)

a Models are adjusted for ethnicity, communities of residence, sex, age, gestational age, and secondhand-smoke exposure.

**Table 10:** Joint effect of *in utero* exposure [OR (95% CI)] to maternal smoking and *GSTP1* Ile105Val genotypes with wheezing outcomes, non-Hispanic and Hispanic whites combined.^a^

		*In utero* exposure to maternal smoking	
Outcome	*GSTP1* Ile105Val genotypes	No	Yes
Lifetime diagnosis of asthma	Ile/Ile	1.0	1.1 (0.7–1.6)
	Ile/Val or Val/Val	1.2 (0.9–1.5)	1.4 (0.9–2.0)
Early-onset asthma	Ile/Ile	1.0	1.7 (0.9–2.9)
	Ile/Val or Val/Val	1.4 (0.9–1.9)	2.0 (1.1–3.3)
Late-onset asthma	Ile/Ile	1.0	0.7 (0.3–1.3)
	Ile/Val or Val/Val	1.1 (0.8–1.5)	1.0 (0.6–1.7)
Current wheezing^b^	Ile/Ile	1.0	1.2 (0.7–1.7)
	Ile/Val or Val/Val	1.0 (0.7–1.1)	1.9 (1.3–2.6)
Medication for wheeze^b^	Ile/Ile	1.0	0.9 (0.5–1.5)
	Ile/Val or Val/Val	1.1 (0.8–1.3)	1.9 (1.2–2.8)

a Models are adjusted for ethnicity, communities of residence, sex, age, gestational age, and secondhand-smoke exposure.

b Significant interaction between *in utero* exposure to maternal smoking and *GSTP1* genotypes (*p* = 0.035 for current wheezing and *p* = 0.043 for medication for wheeze).
